# Virtual Screening of potential drug-like inhibitors against Lysine/DAP pathway of *Mycobacterium tuberculosis*

**DOI:** 10.1186/1471-2105-11-S1-S53

**Published:** 2010-01-18

**Authors:** Aarti Garg, Rupinder Tewari, Gajendra PS Raghava

**Affiliations:** 1 Bioinformatics Centre, Institute of Microbial Technology, Sector-39A, Chandigarh, India; 2 Department of Biotechnology, Panjab University, Chandigarh, India

## Abstract

**Background:**

An explosive global spreading of multidrug resistant *Mycobacterium tuberculosis *(*Mtb*) is a catastrophe, which demands an urgent need to design or develop novel/potent antitubercular agents. The Lysine/DAP biosynthetic pathway is a promising target due its specific role in cell wall and amino acid biosynthesis. Here, we report identification of potential antitubercular candidates targeting *Mtb *dihydrodipicolinate synthase (DHDPS) enzyme of the pathway using virtual screening protocols.

**Results:**

In the present study, we generated three sets of drug-like molecules in order to screen potential inhibitors against *Mtb *drug target DHDPS. The first set of compounds was a combinatorial library, which comprised analogues of pyruvate (substrate of DHDPS). The second set of compounds consisted of pyruvate-like molecules i.e. structurally similar to pyruvate, obtained using 3D flexible similarity search against NCI and PubChem database. The third set constituted 3847 anti-infective molecules obtained from PubChem. These compounds were subjected to Lipinski's rule of drug-like five filters. Finally, three sets of drug-like compounds i.e. 4088 pyruvate analogues, 2640 pyruvate-like molecules and 1750 anti-infective molecules were docked at the active site of *Mtb *DHDPS (PDB code: 1XXX used in the molecular docking calculations) to select inhibitors establishing favorable interactions.

**Conclusion:**

The above-mentioned virtual screening procedures helped in the identification of several potent candidates that possess inhibitory activity against *Mtb *DHDPS. Therefore, these novel scaffolds/candidates which could have the potential to inhibit *Mtb *DHDPS enzyme would represent promising starting points as lead compounds and certainly aid the experimental designing of antituberculars in lesser time.

## Background

Causing massacre especially in Asia and Africa, Tuberculosis (TB) prevalence and mortality rates have probably been mounting globally for last several years [1]. Further, association of TB with HIV patients and emergence of multiple drug-resistant *Mycobacterium tuberculosis *(*Mtb*) to isoniazid and rifampicin and extensive drug-resistant *Mtb *to any floroquinolone, amikacin and capreomycin is a growing alarm. Despondently, more than two million people happen to be victim of TB annually and globally [2-4]. World Health Organization (WHO) 2008 report has mentioned the statistics regarding the occurrence of 9.2 million new cases and 1.7 million deaths from TB in 2006, out of which 0.7 million cases and 0.2 million deaths were in HIV-positive patients [5]. These numbers observed to be boosted compared with those reported by the WHO for the previous years. Therefore, discovery of novel unexploited drug target enzymes and their inhibitors besides generating analogues of existing drugs is a major challenge in the field of drug discovery and designing.

The amino acids play a major role in defining the cellular growth, cell wall and protein synthesis of bacterial system. Importantly, the absence of *de novo *synthesis of protein building blocks and requirement of amino acids as dietary components in mammals implies that specific inhibitors of amino acid biosynthetic pathways would display a novel class of antibacterial agents through inhibition of cell wall and protein synthesis with no mammalian toxicity. For past few years, Lysine/DAP biosynthetic pathway has been gaining high attention due to its foremost feature in the synthesis of D, L-diaminopimelic acid (*meso*-DAP) and lysine. Both components are essential for cross-linking peptidoglycan chains to provide strength and rigidity to the bacterial cell wall [6-8]. It has been observed that *Mycobacterium *cell walls are characterized by an unusual high DAP content. Moreover, gene-knockout experiments with *Mycobacterium smegmatis *has demonstrated the essentiality of DAP pathway for the bacteria, where the absence of DAP results in cell lysis and death [9]. In view of its importance, the designing of potential inhibitors against any enzyme of this pathway may display a novel classes of antitubercular agents.

The present study mainly focused on dihydrodipicolinate synthase (DHDPS) enzyme of the pathway, catalyses the first committed step towards *meso*-DAP formation by condensation of substrate pyruvate with active site residue (LYS-171), which results in the formation of a Schiff-base [10,11]. Next, tautomerisation and aldol type reaction with aspartate *β*-semialdehyde generates an enzyme-tethered acyclic intermediate that undergoes transimination to form heterocyclic [(4*S*)-4-hydroxy-2,3,4,5-tetrahydro-(2*S*)-dipicolinate] (HTPA). The release of HTPA from the active site with elimination of water molecules provides product dihydrodipicolinate (DHDP) [12]. The three-dimensional crystal structures of DHDPS from *Escherichia coli*, *Nicotiana sylvestris, Staphylococcus aureus*, *Mtb*, *Salmonella typhimurium, Bacillus anthracis, Clostridium botulinum, Corynebacterium glutamicum, Thermotoga maritime *and *Bacillus clausii *are available at PDB database. Previously, various structural studies have reported the conservation of active site residues from different bacterial species [13-21].

Till date, designing of inhibitors against DHDPS (mainly from *E. coli*) is being carried out using experimental procedure; however, no potent inhibitors have been reported. However, analogues of pyruvate such as α-ketobutyrate, α-ketoglutarate, glyoxylate and fluoropyruvate have been shown to be competitive inhibitors of DHDPS with respect to pyruvate. Additionally, few inhibitors based on DHDP or HTPA structures showing weak to moderate inhibitory activity is also reported [22-24]. Recently, Mitsakos *et al *[25] has demonstrated that several experimentally known inhibitors displayed a clear differentiation in inhibition of DHDPS enzymes from different bacterial species, hence, suggested that designing of inhibitors against DHDPS should be specific to bacterial species rather than a broad-spectrum inhibitor.

Keeping in view, the importance of DAP pathway in *Mtb *and low outcome of DHDPS inhibitors using experimental procedures, we have made an attempt to screen inhibitors of *Mtb *DHDPS using virtual screening procedures. The present work of screening of DHDPS inhibitors is reported for the first time, hence, would be a great help in aiding the experimental studies and rational development of novel drugs against *Mtb.*

## Methods

### Generation of combinatorial library of pyruvate analogues

**Figure 1:**
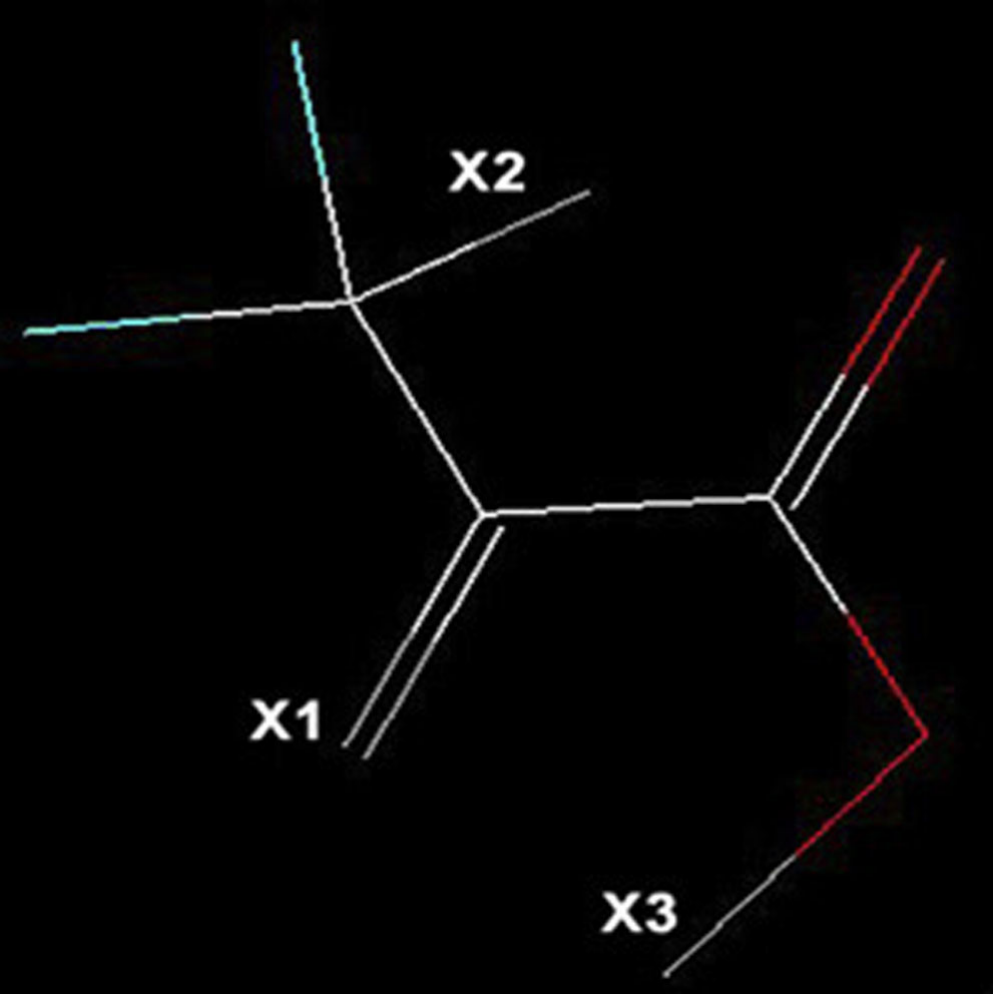
**Pyruvate template with three substitution sites used for the combinatorial library generation**.

The LeadGrow module of VLifeMDS [26] provides facility for the generation of combinatorial library starting with a template structure (also called as core/scaffold) to which the side chains are attached/substituted from the list of given substituents. Thus, if X1 and X2 are two substitution sites on the template, where X1 has L and X2 has M choices for the attachment/substitution of atoms/groups, then a library with L × M number of compounds can be generated. In the present study, the substrate pyruvate was used as a template for the library generation and three substitution sites namely X1, X2, X3 were chosen as shown in Figure 1, where the atoms/groups can be substituted. For X1 site, the substitutions were carried using non-toxic element such as N, O, F, P, S, Cl, Br, I, whereas for X2 and X3 the alkyl (ethyl, isopropyl, butyl, isobutyl, tertiary butyl, methyl), alkene (vinyl, allyl), acids (carboxyl, carbonic), ketone (methylketone), carbomethoxy (acetate, carbomethoxy), charged (carboxylate), and others (amine, carbonyl, -O-CH_3_, -OC_2_H_5_, amide, cynide, cynate, isocynate, -C = N, -N = C, azo, hydrazo, nitroso, nitro, sulfone, -S = OOO, oxime, CH_2_-C = N) subsitutions were applied. The generated combinatorial library was subjected to Lipinski's rule of five filters [27] to get drug-like orally-bioavailable compounds. Finally, we were left with 4088 pyruvate analogues to be docked for the screening of potential inhibitors against DHDPS.

### Pyruvate-like molecules

Since the similarities in structures are indicative of similarities in bioactivities, therefore, structure based searching of databases/libraries has been gaining high demand nowadays [28,29]. In the present study, VLifeMDS package was used for carrying out 3D flexible search (using pyruvate as a template) against most popular databases i.e. National cancer Institute (NCI) Open database with ~2,60,071 compounds, and a library of sub/superstructures of pyruvate from PubChem database which constituted 21,061 compounds. These databases were first subjected to Lipinski's rule of five constraints (mentioned earlier), which reduced their size to ~160,000 and 17,157 compounds respectively. The searching was based on 3D superimposition, where the query pyruvate and the compounds present in the databases were structurally superimposed for rmsd calculations, in order to check whether the atoms in the match mapping meets the spatial constrains (distance, angle, dihedral) in a query or not.

The databases generally store only a single low-energy conformation or a limited number of conformations for each compound, which may lead to the reduction in the hit rates. Therefore to make the search more effective, the 3D flexible search was carried out in the present study. Here, the conformers for each compound was generated using Metropolis Monte Carlo simulations, which explores the compound's conformational search space using random moves by altering torsion angle values. Here, the tolerance limit (which defines the rmsd cut-off) was set to 50%, such that the hits with rmsd value greater than 50% were discarded. Following the 3D search, 291 and 2349 compounds from NCI and PubChem libraries were retrieved. Finally, we got total 2640 molecules, which are structurally similar to pyruvate molecules. In this study, these molecules will be called pyruvate-like molecules.

### Anti-infective molecules

Additionally, 3847 anti-infective compounds, consisting of 1743 antibacterials were retrieved from PubChem database. Out of 3847, only 1750 anti-infectives satisfied the Lipinski's rule of five constraints. These compounds were highly diverse from the pyruvate such that none of the anti-infectives showed 2D/3D similarity with pyruvate. Hence, the docking of these compounds would help to screen the diverse classes of antitubercular agents against *Mtb *DHDPS.

### Ligand-receptor flexible docking

To find the binding affinities between target receptor and screened out compounds, an automated flexible docking of ligands at the flexible active site of receptor was carried out using AutoDock (v.4.0) software [30]. The software facilitates the internal degree of freedom along with the values of translation and rotation for the side chains of selected active residues as well as for the ligands in search of its suitable bound conformations. Undoubtedly, introduction of flexibility makes the docking process computationally more expensive but more superior than rigid ligand-receptor docking. Before docking process, several separate pre-docking steps: ligand preparation, receptor preparation and grid map calculations were performed. The ligand and receptor preparation stage involved the addition of hydrogen atoms, computing charges, merging non-polar hydrogen atoms and defining AD4 atom types to ensure that atoms conformed to the AutoDock atom types. Next, information about rotatable torsion bonds that defines the bond flexibility was acquired. The ligands and receptor molecule preparation was followed by grid construction using AutoGrid module. During grid construction, atom types of the ligand, which acted as probes in the calculation of grid maps, were identified. The grid with default volume of 40 × 40 × 40 Å with a spacing of 0.375 Å centered on the receptor was prepared. For conformational search, the docking calculations using the genetic algorithm (GA) procedure with default parameters was performed. The GA computed the fitness of a docked candidate every time by measuring the minimum values of free energy binding (ΔG) based on different types of energy evaluations. In the present study, the python scripts were used for carrying out automated docking process.

## Results and discussion

In the present study, different virtual screening approaches were used for selecting potential inhibitors against *Mtb *DHDPS. The first approach employed the generation of combinatorial library i.e. analogues of pyruvate. In the second approach pyruvate-like molecules generated using 3D flexible similarity search against available databases/libraries. Thirdly, to screen diverse classes of antitubercular agents, drug-like 1750 anti-infectives available at PubChem database were retrieved. Finally, these three sets of compounds i.e. generated pyruvate analogues, pyruvate-like molecules and anti-infectives were docked into the active site of receptor with the purpose of sorting potential inhibitors of *Mtb *DHDPS.

The three-dimensional crystal structure of *Mtb *DHDPS stored in the PDB file (code: 1XXX) [20] was obtained from protein databank. *Mtb *DHDPS is a homotetramer and each subunit with 300 amino acid residues comprises: N-terminal (β/α)_8_-barrel domain (residues 1-233) and a C-terminal domain (residues 234-300), which consists of three α-helices. The residues bounded the active site are THR54, THR55 TYR143, ARG148 and LYS171. Particularly, LYS171 responsible for substrate binding and catalysis are located at the centre of each monomer in the (b/α)_8_-barrel domain facing the central cavity of the tetramer. In *E. coli *DHDPS enzyme the equivalent residue is LYS161. Using PYMOL software, all the 1587 water molecules, eight DTT molecules, eight Mg^2+ ^and eight Cl^- ^ions were removed.

### Docking of pyruvate analogues

**Figure 2:**
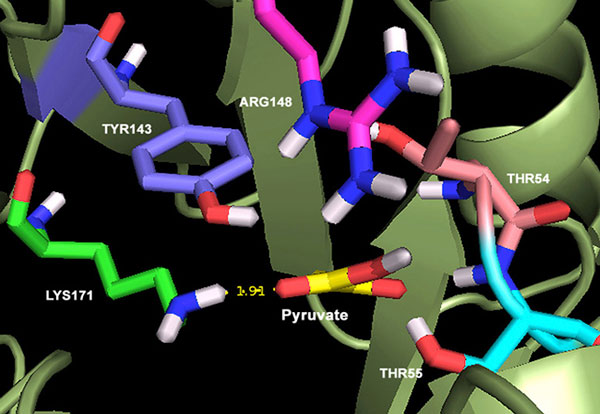
**Docking of pyruvate to the active site of Mtb DHDPS**.

First, 4088 drug-like pyruvate analogues were docked into the active site of *Mtb *DHDPS. For the validation of docking process, the substrate pyruvate was also introduced as a control with the purpose of screening the compounds with docking score greater than the substrate. The detailed view of docking of pyruvate to the active site is shown in Figure 2, which exhibited molecular docking with ΔG value of -6.31 (kcal/mol). The pyruvate lying near the vicinity of LYS171 observed to be forming a hydrogen bond with side chain of LYS171-NH2. Additionally, we also introduced 5 experimentally known inhibitors i.e. Piperidine-2,6-dicarboxylic acid (43%), Dimethylpiperidine-2,6-dicarboxylate (24%), Pyridine-2,6-dicarboxylic acid (75%), 1,4-dihydro-4-oxopyridine-2,6-dicarboxylic acid (73%) and Dimethyl-1,4-dihydro-4-oxopyridine-2,6-dicarboxylate (84%) of *Mtb *DHDPS, inhibiting the enzyme activity in the range of 24-84% [25]. These inhibitors were mainly the analogues of the product DHDP. The docking ΔG values (i.e. -6.38, -4.98, -7.67, -7.60, and -6.39 kcal/mol respectively) ranked four of these inhibitors correctly with respect to their percent inhibition (%) values. Additionally, it was found that four inhibitors were docked with better ΔG values than substrate, however only one inhibitor (Dimethylpiperidine-2,6-dicarboxylate) which caused poor 24% inhibition of the enzyme actvity exhibited poor docking in comparison with substrate and other inhibitors. Another inhibitor, Pyridine-2,6-dicarboxylic acid, which caused 75% inhibition in the enzyme activity provided ΔG value of -7.67 (kcal/mol).

**Figure 3:**
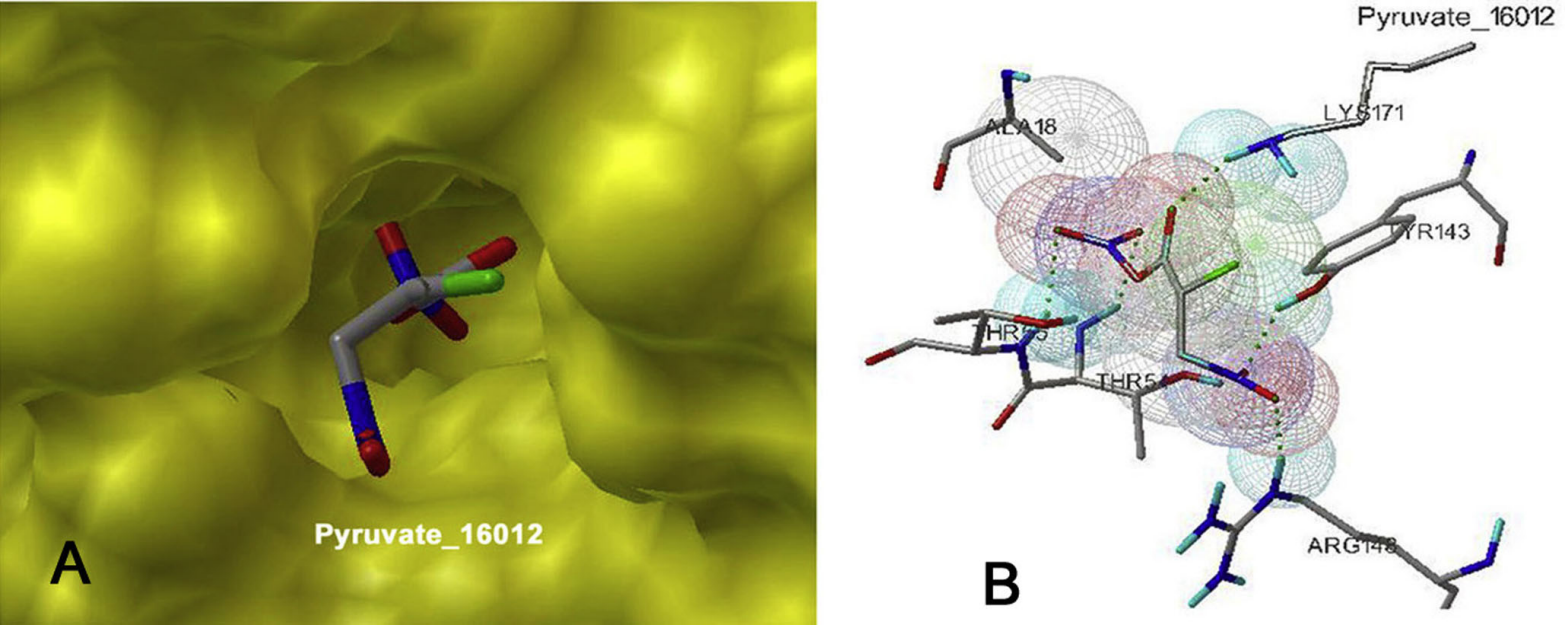
**Docking of pyruvate analogues**. Pyruvate analogue lying deep into the binding pocket (A) of *Mtb *DHDPS by establishing bonded and non-bonded interactions (B).

**Figure 4:**
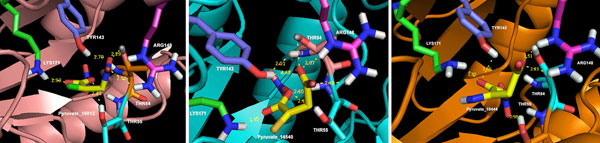
**Binding poses for top three pyruvate analogues**.

For pyruvate analogues, ΔG values in the range of -9.07 to -3.37 (kcal/mol) was obtained. Approximately 374 compounds were found to be docked with ΔG values better than pyruvate. Importantly, top 10 hits exhibited strong binding by yielding free energy binding values superior than -8.43 (kcal/mol) (Table 1). Further, we carried out detailed analysis of the top 10 docking hits in terms of their structures and ligand-receptor interactions. The top ligands were found to be lying deep into the binding cavity exhibiting all major interactions such as hydrogen bonding, Van der Waals (VDW) and hydrophobic (shown in Figure 3A and 3B). Nevertheless, hydrogen bonding with LYS171 as well as with other conserved active site residues was found to be dominating. For instance, the predicted inhibitors formed hydrogen bonds with LYS171-NH2, ARG148-NH2, TYR143-OH, THR54-OH and THR55-OH. Here, we have shown the docking poses of top 3 compounds (or their conformers) i.e Pyruvate_16012, Pyruvate_14540 and Pyruvate_10444 (Figure 4), revealing the binding of these compounds at the active site much better than substrate and experimentally known inhibitors. Though, the importance of ARG148 has not been elucidated in *Mtb *DHDPS, however, the conserved equivalent residue i.e. ARG138 from *E. coli *DHDPS has been demonstrated to be playing imperative role in binding, specifically feed back inhibition for (*S*)-lysine. Hence, the present observation i.e. involvement of ARG148 in binding with few potential inhibitors could be further exploited experimentally. The structures of top 10 compounds docked with highly favorable scores i.e. more negative values than substrate receptor complex are also given in Table S1 (see Additional file 1). Most of these hits hold the chemical structure feature group such as oxime, sulfone, carboxylate, carbonic, carboxyl, nitroso, and nitro. Furthermore, we have also calculated the correlation between molecular weight and ΔG values for these top hits. However, very low correlation value of -0.28 was obtained which indicated that the predicted binding affinity was mainly due to specificty and not due to molecular size.

**Table 1:** Top 10 pyruvate analogues with better free binding energy values in comparison with substrate

S.No	Inhibitors	ΔG values (kcal/mol)	Molecular Weight
1.	Pyruvate_16012	-9.07	197.516

2.	Pyruvate_14540	-8.94	192.133

3.	Pyruvate_10444	-8.87	175.082

4.	Pyruvate_14988	-8.81	194.129

5.	Pyruvate_13516	-8.62	192.049

6.	Pyruvate_10892	-8.60	177.078

7.	Pyruvate_12332	-8.56	180.073

8.	Pyruvate_14380	-8.54	193.141

9.	Pyruvate_13658	-8.50	207.084

10.	Pyruvate_11915	-8.43	162.063

### Docking of pyruvate-like molecules

The virtual screening using 3D similarity based search could provide two main advantages- i) helps to narrow down the size of large databases/libraries to be screened, which eventually reduces the computational time required to dock each library compound and ii) 3D searching retrieves the compounds, out of which some are the same class of compounds to which query belongs, but some others may be entirely new classes of compounds, which may directly lead to the discovery of novel lead compounds.

**Figure 5:**
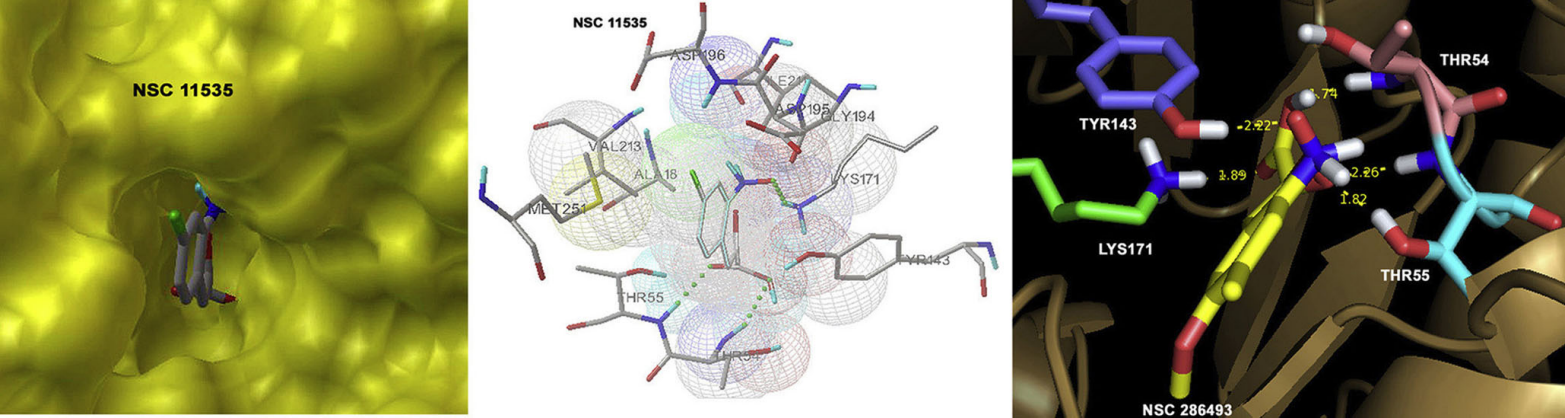
**Docking of pyruvate-like NCI compounds**. Top predicted inhibitors from NCI databases establishing interactions with active site residues of *Mtb *DHDPS.

The docking of 291 and 2349 pyruvate-like compounds (structurally similar to pyruvate) retrieved from NCI and PubChem was carried out using the same docking protocol as mentioned earlier for pyruvate analogues. For NCI library, 50 pyruvate-like compounds provided free energy binding values better than pyruvate control, wheras 8.9% of the compounds were not able to fit perfectly into the binding pocket, yielding positive free energy binding values. The ΔG values in the range of -9.03 to -8.16 (kcal/mol) and ids of the top 10 NCI compounds are summarized in the Table 2. The structures of these hits are given in Table S2 (see Additional file 1). All these top hits were characterized by aromaticity along with presence of heteratoms i.e., N, Cl, Br in few compounds. These detailed analysis revealed that these 10 inhibitors fit very well into the binding pocket by establishing bonded and nonbonded interactions with active site residue as shown in Figure 5. Similar to the docking of pyruvate analogues, hydrogen bonding was found to be dominant interactions with LYS171, TYR143, THR54 and THR55. Furthermore, the correlation between molecular weight and ΔG values for these 10 inhibitors yielded lower value of -0.55.

**Table 2:** Free binding energy values and structures for top ten NCI hits

S.No	Inhibitors	ΔG values (kcal/mol)	Molecular Weight
1.	NSC 11535	-9.03	291.08

2.	NSC 286493	-8.78	256.24

3.	NSC 5598	-8.76	212.19

4.	NSC 115134	-8.61	226.21

5.	NSC 105301	-8.53	246.63

6.	NSC 62754	-8.43	245.06

7.	NSC 62757	-8.35	166.14

8.	NSC 157880	-8.32	224.20

9.	NSC 62753	-8.16	206.20

10.	NSC 139986	-8.16	246.63

**Figure 6:**
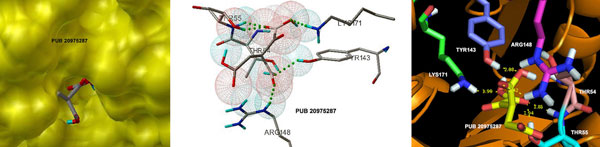
**Docking of pyruvate-like PubChem compound**. Interactions established by the best docked PubChem compound (PUB 20975287).

The docking ΔG values for top 5 pyruvate-like compounds of PubChem are listed in Table 3. Out of 2349, only 250 compounds exhibited docking with free binding energy values better than the substrate. The tops hits were mainly aromatic, however only one compound (PUB20975287) was found to be aliphatic, which displayed strong binding by yielding ΔG value of -8.40 (kcal/mol). The interactions of these inhibitors with active site residue were mainly through hydrogen bonding, VDW and hydrophobic as shown in Figure 6 for the best docked compound i.e. PUB20975287. The structures of these top 5 hits are given in Table S3 (see Additional file 1)

**Table 3:** Free binding energy values and structures for top five PubChem compounds

S.No	Inhibitors	ΔG values (kcal/mol)	Molecular Weight
1.	PUB 20975287	-8.40	190.11

2.	PUB19751056	-8.36	197.15

3.	PUB240601	-8.27	197.15

4.	PUB15288093	-7.91	179.14

5.	PUB18799166	-7.90	179.14

### Docking of anti-infectives

**Figure 7:**
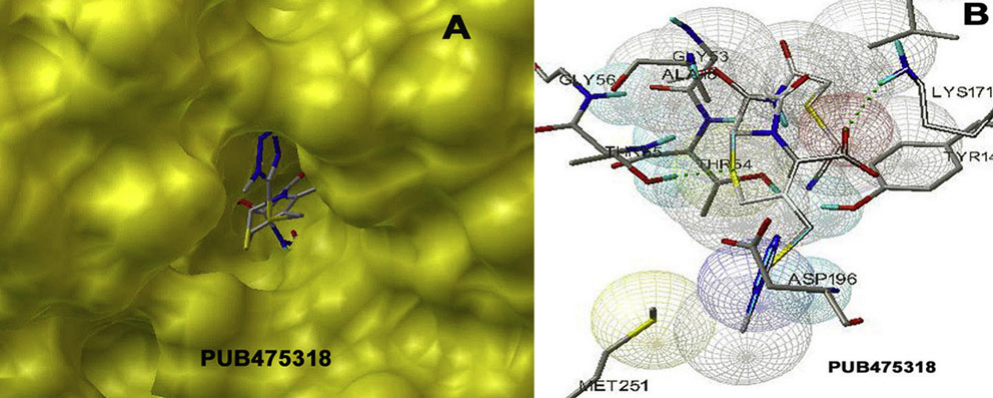
**Docking of top anti-infective PUB475318 lying deeply into the active binding site**.

**Figure 8:**
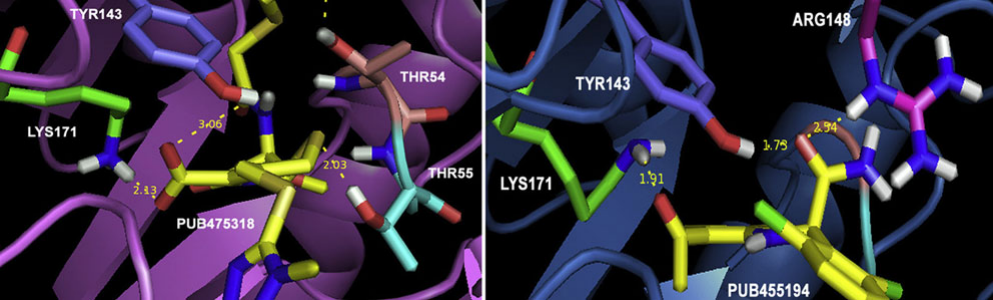
**Docking poses for top two anti-infectives establishing hydrogen bonding at the active site**.

Besides, docking of very diverse 1750 anti-infectives structures was also carried out. Interestingly, 28.5% of the compounds provided positive ΔG values, revealing their inadequacy in adjusting into the binding pocket comfortably. Most of the remaining compounds though provided negative ΔG values, however lower than the substrate, hence, did not fit well in DHDPS binding pocket. Very few 25 anti-infectives provided ΔG values better than substrate and experimentally known inhibitors. Interestingly, the top 2 anti-infectives though provided -29 and -23 kcal/mol of ΔG values; however these failed to establish any interactions with LYS171. Table 4 and Table S4 (Additional file 1) show the free energy binding values and structures for the next top 5 compounds respectively. The first two compounds with ΔG values -12.3 and -10.2 (kcal/mol) were assumed to be the best and potent inhibitors of DHDPS screened out in the present study. The top PUB475318 was able to fit comfortably in the binding pocket of DHDPS establishing bonded and non-bonded interactions very well as shown in Figure 7. The anti-infective PUB475318 is known by the name Cefmetazole, a semisynthetic cephamycin antibiotic with a broad spectrum of activity against both gram-positive and gram-negative microorganisms [31]. The compound was also explored during the high throughput screening for the identification of inhibitors against *Mtb *H37Rv [32]. On the other hand, PUB455194, which was ranked second during the screening process, is known as antiviral agent inhibiting virion. We have also shown in Figure 8, the docking poses of these top 2 anti-infectives establishing hydrogen bonding at the active site of DHDPS. Hence, docking of anti-infectives helped us in the identification of compounds with more diverse topology in comparison to pyruvate analogues or pyruvate-like molecules.

**Table 4:** Docking results for top five anti-infectives retrieved from PubChem database

S.No	Inhibitors	ΔG values (kcal/mol)	Molecular Weight
**1**.	**PUB475318**	**-12.34**	**470.54**

**2**.	**PUB455194**	**-10.21**	**351.23**

3.	PUB4451056	-8.58	139.028

4.	PUB3092	-8.31	227.18

5.	PUB702695	-8.29	184.13

## Conclusion

To conclude, we have employed several virtual screening protocols such as generation of combinatorial library, 3D flexible search and molecular docking to identify potential inhibitors against *Mtb *DHDPS. Several potential drug-like inhibitors have been screened out showing strong binding affinity to *Mtb *DHDPS. Additionally, few anti-infectives with highly diverse topology from the pyruvate also displayed strong binding. Though experimental studies are indispensable to mark them as lead compound for the development of novel drugs against *Mtb*, however, screened out inhibitors would undoubtedly aid the experimental designing of antitubercular agents expeditiously.

## List of abbreviations used

The abbreviations used are: DAP: Diaminopimelic Acid; LYS161: Lysine-161; *Mtb: Mycobacterium tuberculosis*; DHDPS: Dihydrodipicolinate synthase; TB: Tuberculosis; DHDP: Dihydrodipicolinate; NCI: National cancer Institute

## Competing interests

The authors declare that they have no competing interests.

## Authors' contributions

AG, GPSR and RT conceived and designed the experiments. AG performed the experiments and wrote perl scripts. GPSR and AG analyzed the data. AG wrote the manuscript. AG, RT and GPSR carried out revision of the manuscript. All authors have seen and approved the manuscript.

## Supplementary Material

Additional file 1 Four tables (Table S1-S4) giving: structures of top 10 pyruvate analogues hits (Table S1), structures of top ten NCI hits (Table S2), structures for top five PubChem compounds (Table S3) and structures of top five anti-infectives (Table S4). All the tables are provided as a single word document. Click here for file
