# Supplementary material for: Virtual Screening of potential drug-like inhibitors against Lysine/DAP pathway of Mycobacterium tuberculosis
Source: BMC Bioinformatics. 2010 Jan 18;11(Suppl 1):S53. doi: 10.1186/1471-2105-11-S1-S53 (PMC3009526; doi:10.1186/1471-2105-11-S1-S53)
Supplement: Additional file 1 — Four tables (Table S1-S4) giving: structures of top 10 pyruvate analogues hits (Table S1), structures of top ten NCI hits (Table S2), structures for top five PubChem compounds (Table S3) and structures of top five anti-infectives (Table S4). All the tables are provided as a single word document. [file 1471-2105-11-S1-S53-S1.doc]

**Table S1 – Structures of t**op 10 pyruvate analogues

| **S.No** | **Inhibitors** | **Structures** |
| --- | --- | --- |
|  | Pyruvate_16012 | 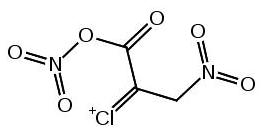 |
|  | Pyruvate_14540 | 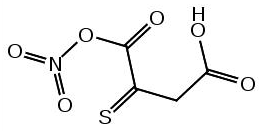 |
|  | Pyruvate_10444 | 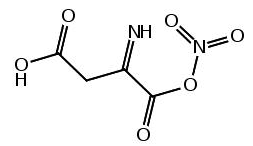 |
|  | Pyruvate_14988 | 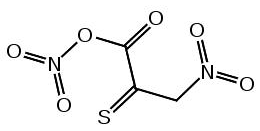 |
|  | Pyruvate_13516 | 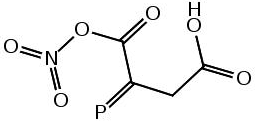 |
|  | Pyruvate_10892 | 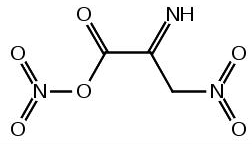 |
|  | Pyruvate_12332 | 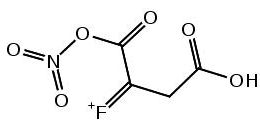 |
|  | Pyruvate_14380 | 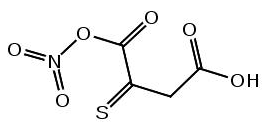 |
|  | Pyruvate_13658 | 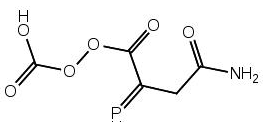 |
|  | Pyruvate_11915 | 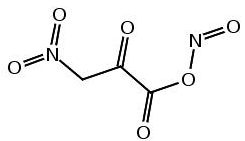 |

**Table S2 - S**tructures for top ten NCI hits

| **S.No** | **Inhibitors** | **Structures** |
| --- | --- | --- |
|  | NSC 11535 | 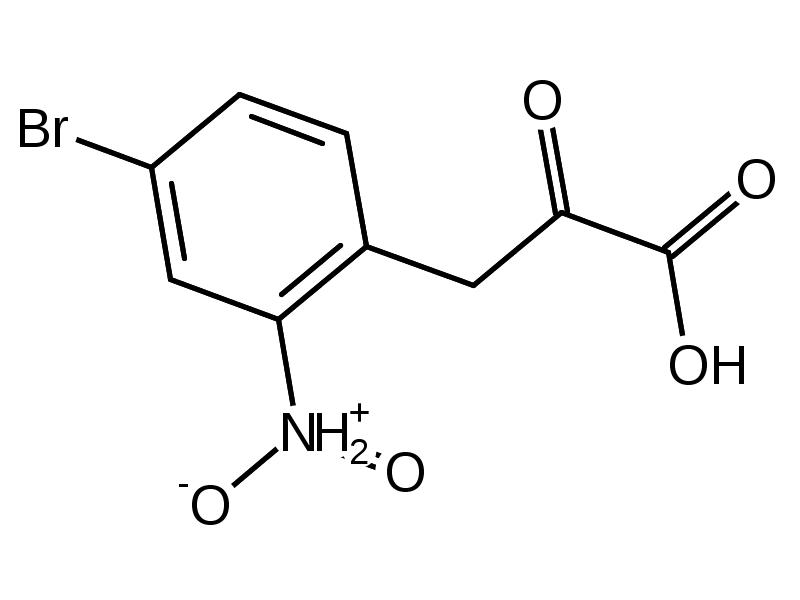 |
|  | NSC 286493 | 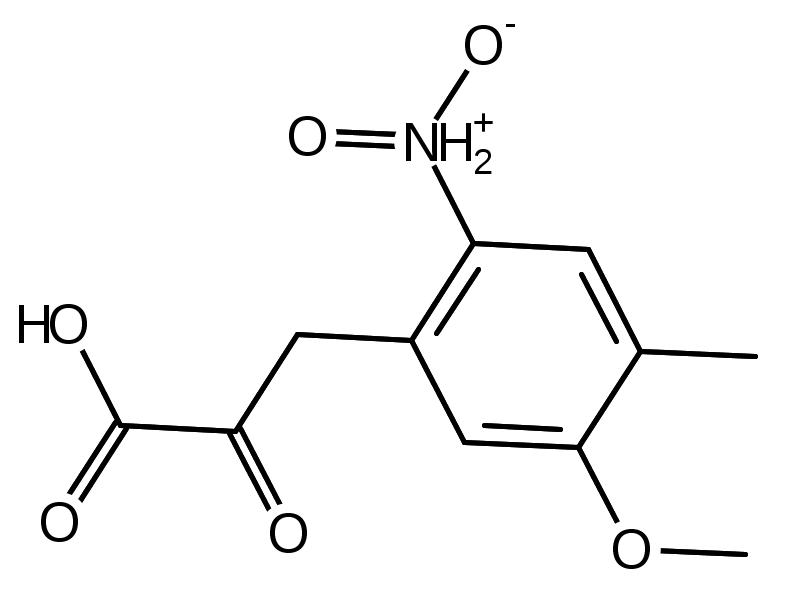 |
|  | NSC 5598 | 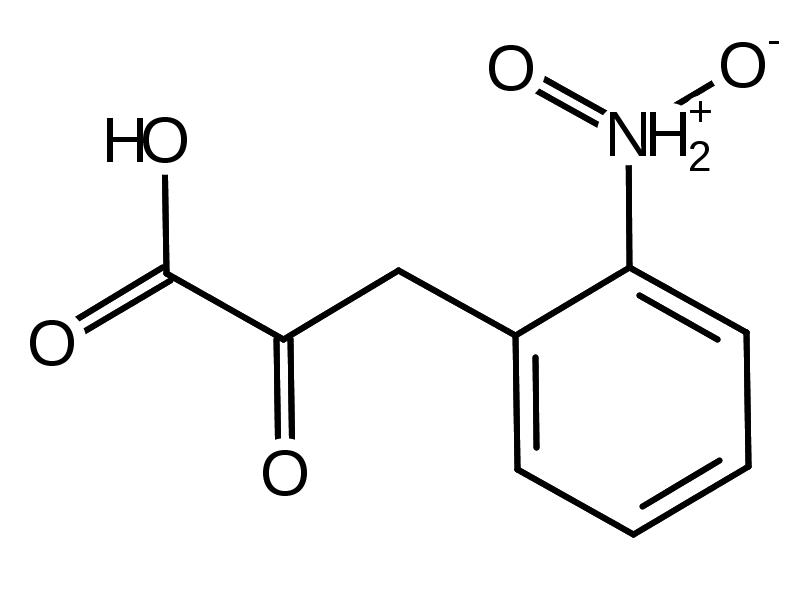 |
|  | NSC 115134 | 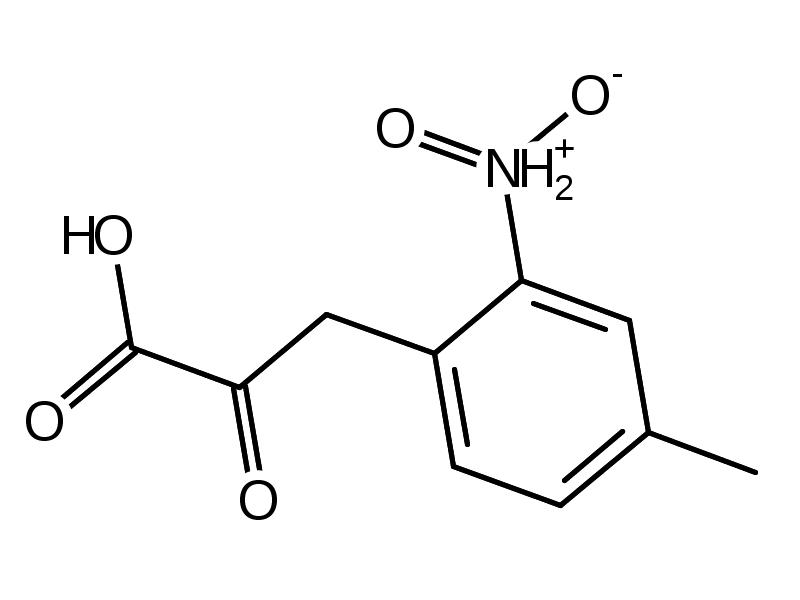 |
|  | NSC 105301 | 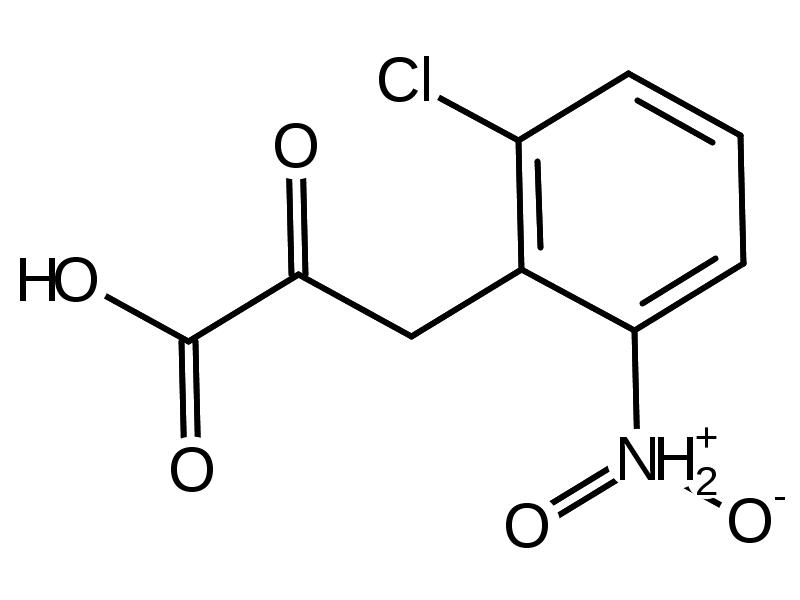 |
|  | NSC 62754 | 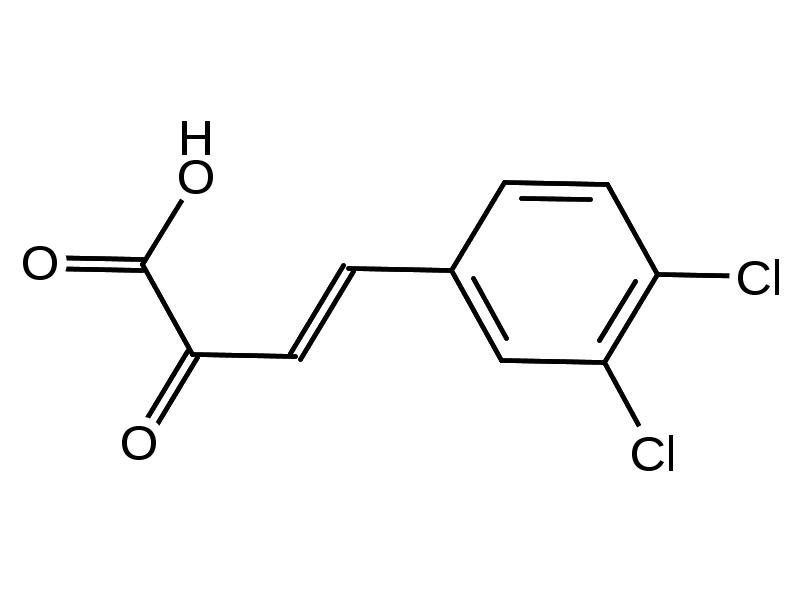 |
|  | NSC 62757 | 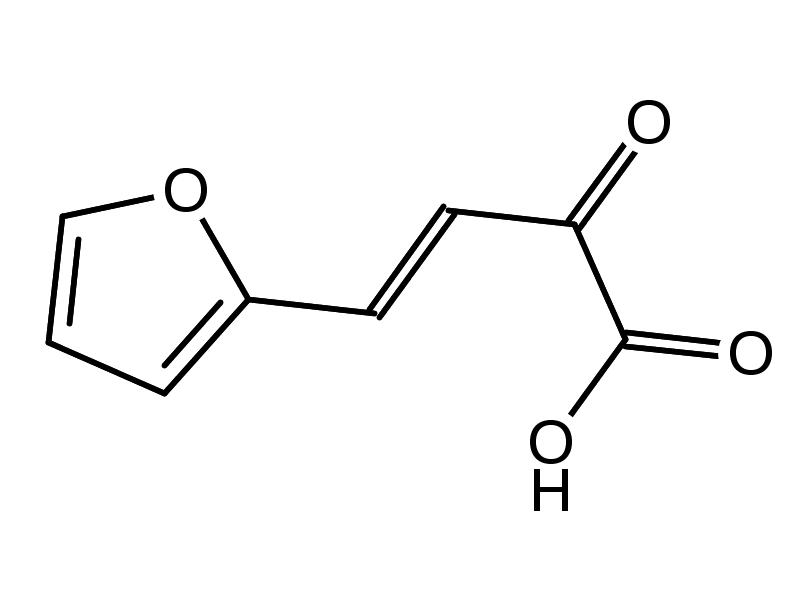 |
|  | NSC 157880 | 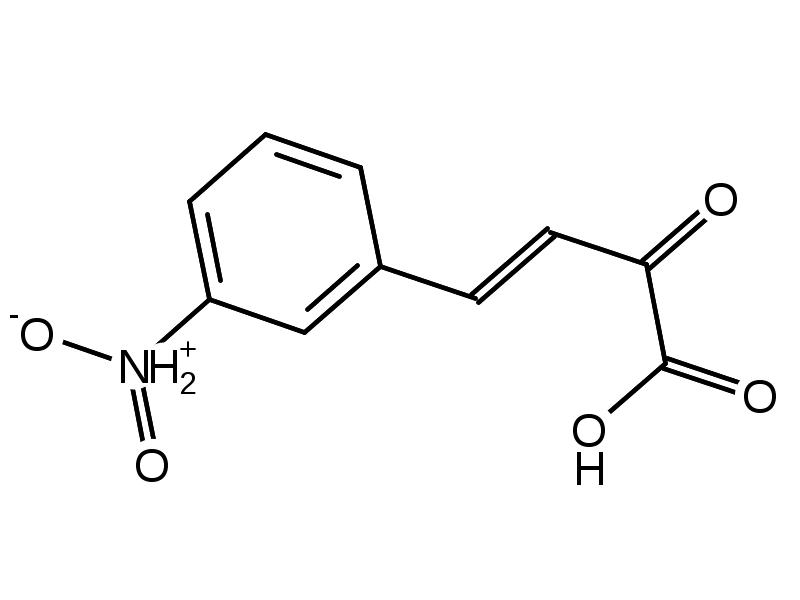 |
|  | NSC 62753 | 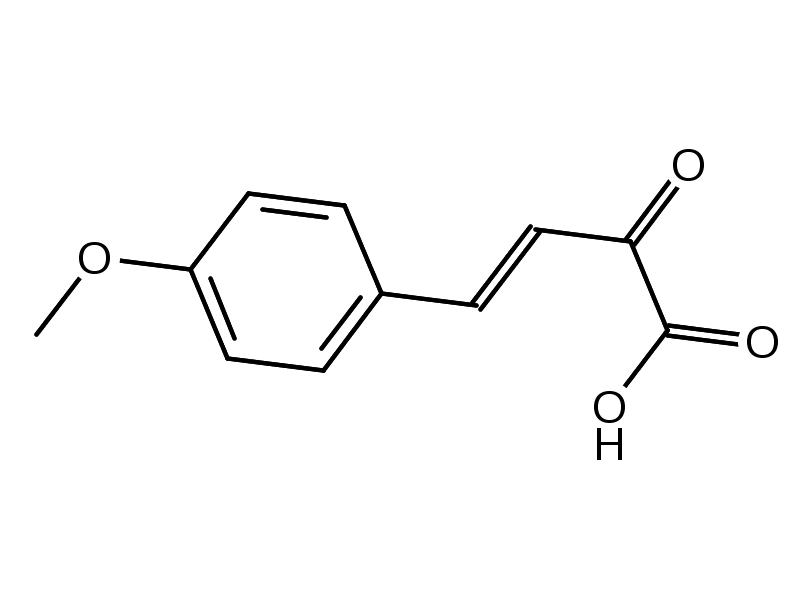 |
|  | NSC 139986 | 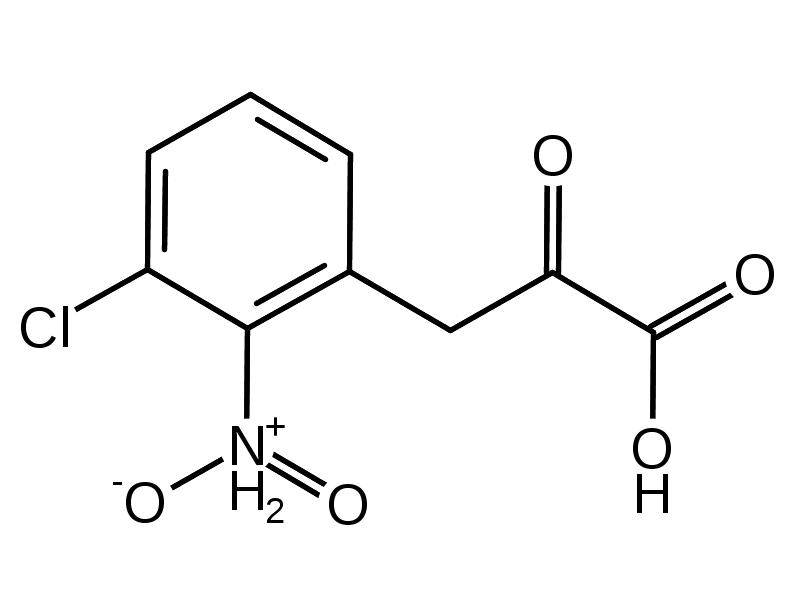 |

**Table S3 - S**tructures for top five PubChem compounds

| **S.No** | **Inhibitors** | **Structures** |
| --- | --- | --- |
|  | PUB 20975287 | 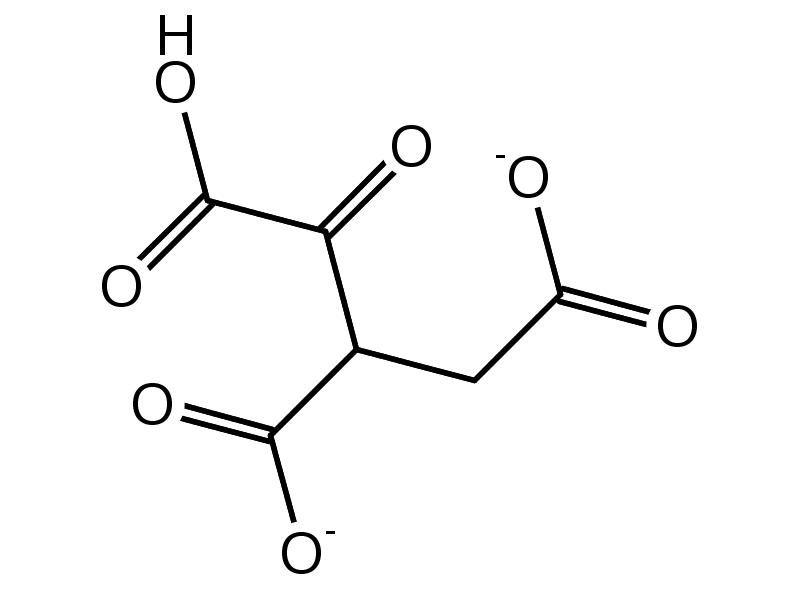 |
|  | PUB19751056 | 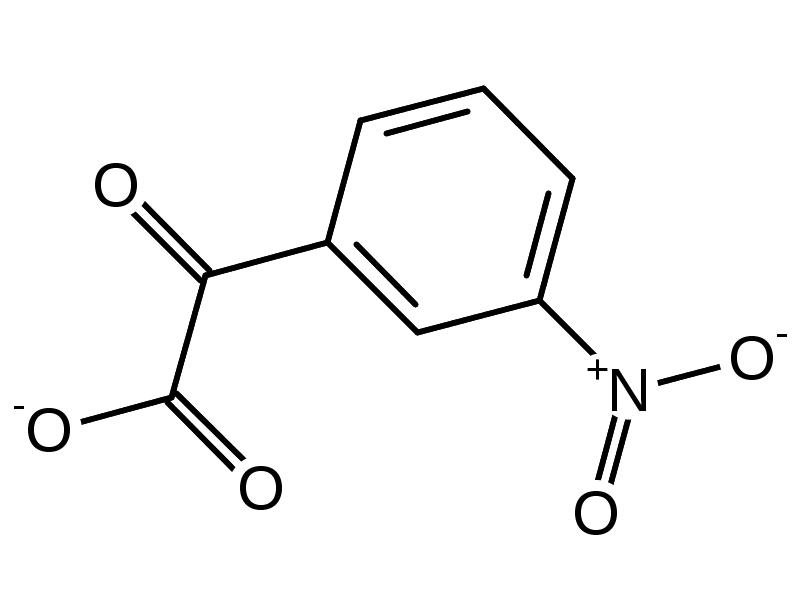 |
|  | PUB240601 | 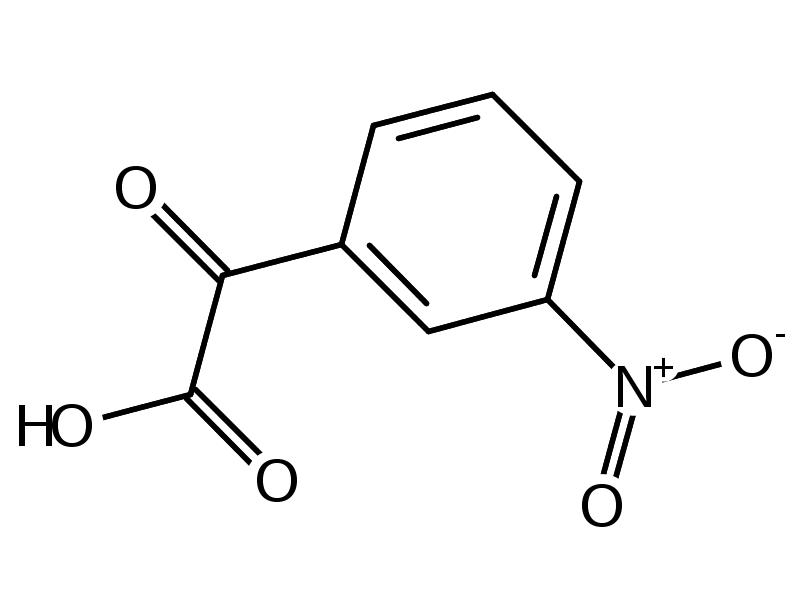 |
|  | PUB15288093 | 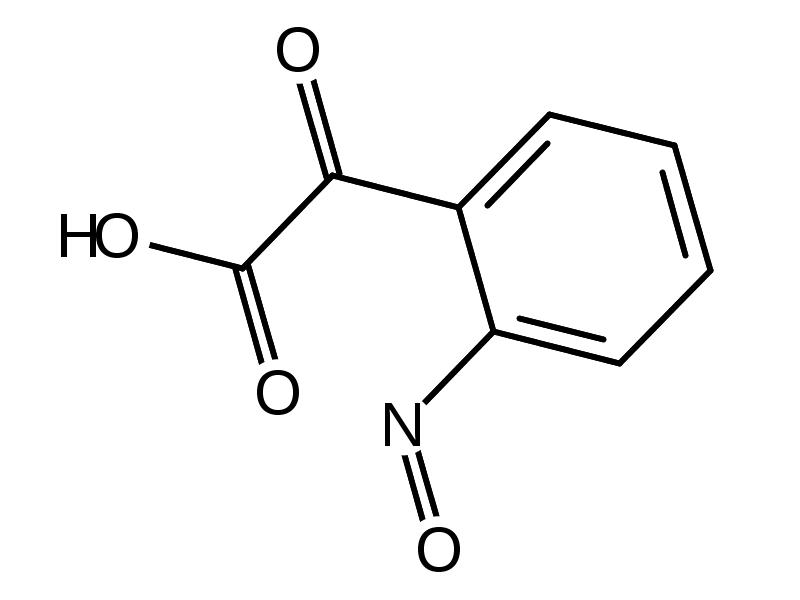 |
|  | PUB18799166 | 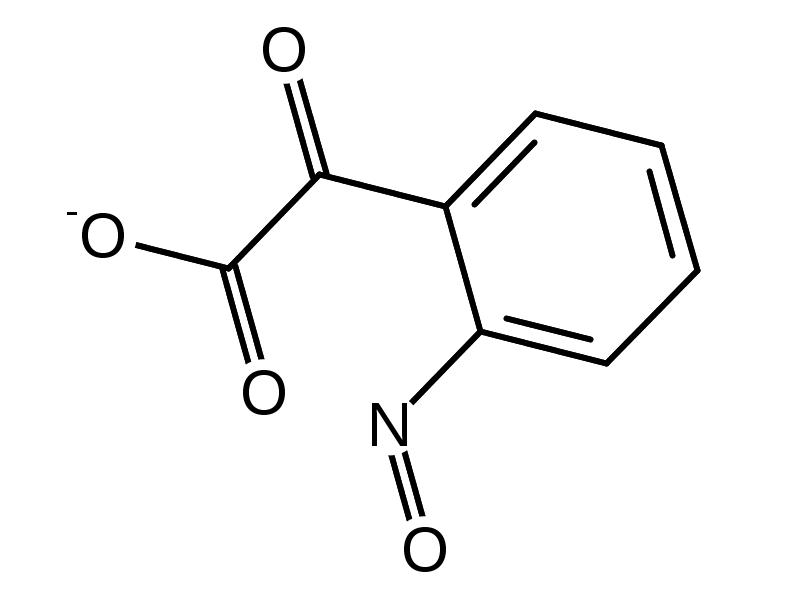 |

**Table S4 – Structures** for top five anti-infectives

| **S.No** | **Inhibitors** | **Structures** |
| --- | --- | --- |
|  | **PUB475318** | **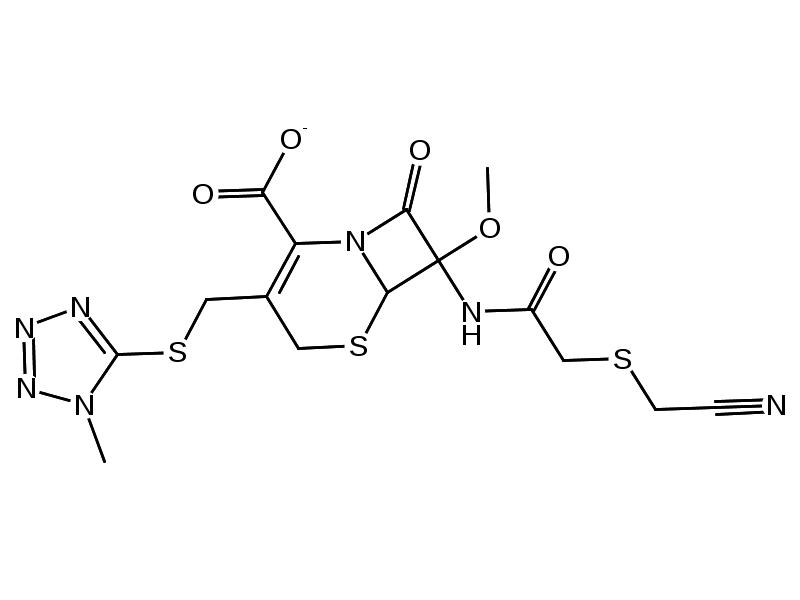** |
|  | **PUB455194** | **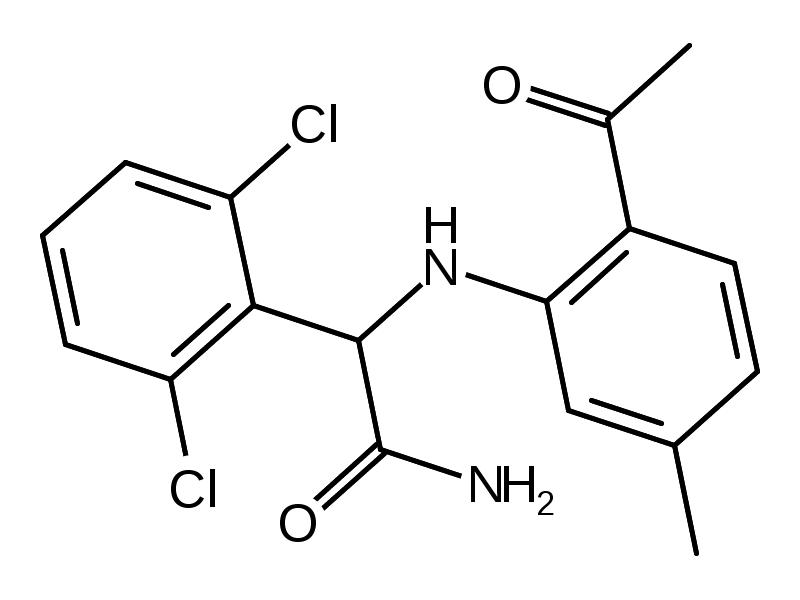** |
|  | PUB4451056 | 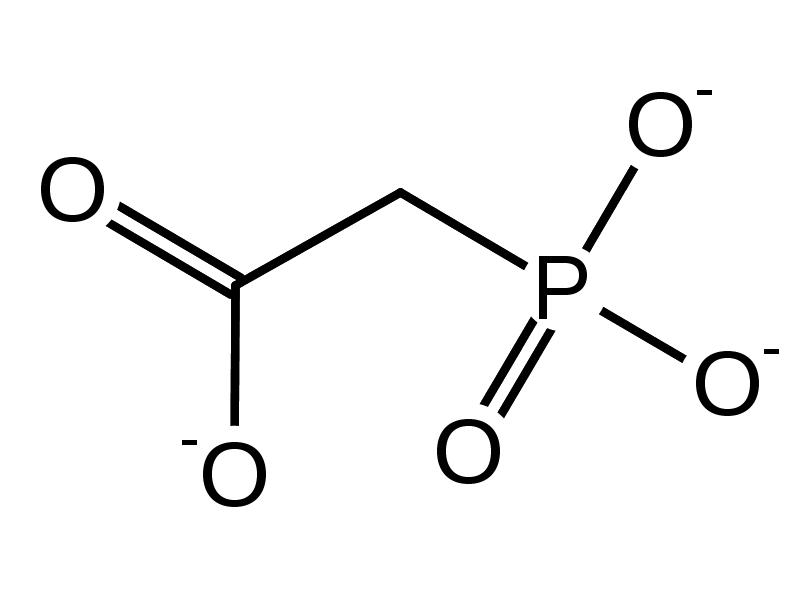 |
|  | PUB3092 | 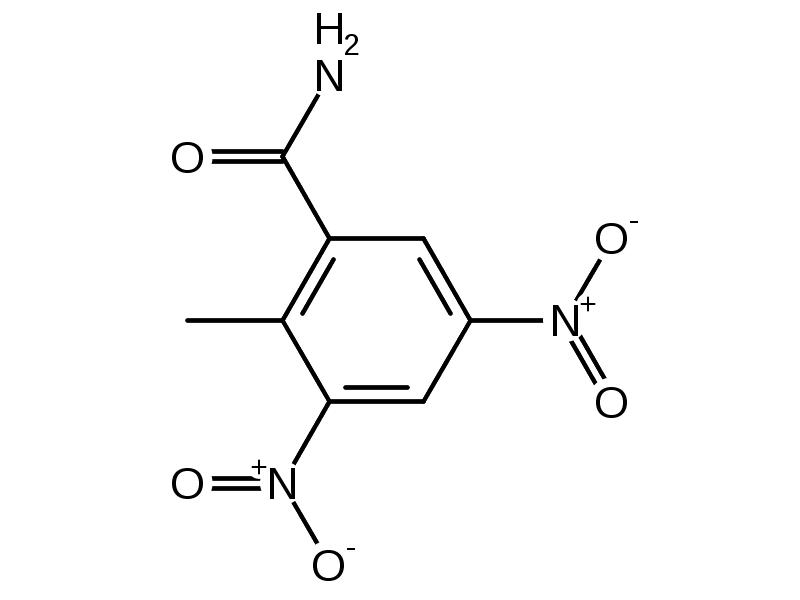 |
|  | PUB702695 | 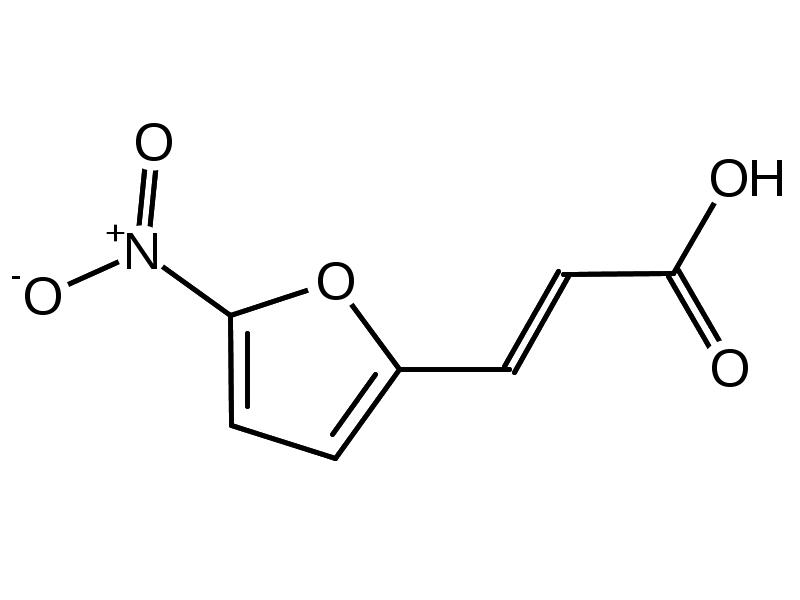 |
